# Nitrous-Oxide Oxygen; Its Field and Limitations in Surgery and Obstetrics

**Published:** 1917-06

**Authors:** W. Hamilton Long

**Affiliations:** Fellow of the American Association of Anesthetists; Member and Ex-President (1915-1916) Interstate Association of Anesthetists, and the Louisville Society of Anesthetists; Supervising Anesthetist (Visiting) Louisville Public Hospital; Editor Department of Anesthetics, Kentucky State Medical Journal


### Nitrous-Oxide Oxygen; Its Field and Limitations in Surgery and Obstetrics.

BY W. HAMILTON LONG, M. D., FELLOW OF THE AMERICAN ASSOCIATION OF ANESTHETISTS; MEMBER AND EX-PRESIDENT (1915-1916) INTERSTATE ASSOCIATION OF ANESTHETISTS, AND THE LOUISVILLE SOCIETY OF ANESTHETISTS; SUPERVISING ANESTHETIST (VISITING) LOUISVILLE PUBLIC HOSPITAL; EDITOR DEPARTMENT OF ANESTHETICS, KENTUCKY STATE MEDICAL JOURNAL.

Nitrous-oxide oxygen is an anesthetic of such value that the modern anesthetist, equipped for his specialty, finds it indispensable. It has, however, to my mind very definite limitations. By using ether in conjunction with it, many of its shortcomings may be overcome, but such an anesthetic is not a nitrous-oxide oxygen administration, and cannot correctly be so classed. We will confine ourselves in this brief article to the "straight" nitrous-oxide oxygen anesthesia and analgesia.

Without going into the history of its general use, so widespread during the past decade, it may be said that its application was confined almost entirely to dentistry for many years, and it seems strange that its obvious advantages were not recognized by surgeons many years ago. Its use for a long time was, as has been said, almost entirely limited to teeth extraction, and to such minor surgical procedures as needed but the most transient narcosis. The possibility and feasibility of its continued or prolonged administration seemed to be overlooked—an agent wherewith unconsciousness could be induced but not maintained; and so transient in its effect that complete recovery usually took place in from thirty seconds to one minute after administration stopped. Thus the sustained anesthesia achieved by an absolutely continuous adminstration was a revelation to us when we first saw it.

Considering the field for this agent in general surgery, we must bear in mind the characteristics of its action, and the points wherein it differs from chloroform and ether. Nitrous oxide must be given by an absolutely "closed" method. All outside air must be excluded, and not only is this essential to successful administration, but rebreathing is usually practiced to insure a more even continuation of the narcosis at its greatest depth, and also to conserve the gas, the cost of which is considerable. Following such a technic with $N_2O$ alone, it is obvious that a fatal asphyxia would soon supervene, hence, for any prolonged administration, a continuous supply of fresh oxygen must be added to the gas. This dilution of the latter with the right amount of oxygen is really what makes nitrous oxide a feasible agent for producing in selected cases a satisfactory working anesthesia—an anesthesia characterized by total unconsciousness without voluntary muscular action, without spasm or cyanosis, but also without the marked relaxation and flaccidity of the abdominal muscles that most surgeons demand and that can be so easily achieved with chloroform and with ether. Its action is especially pretty in cases where a general debilitated condition exists; where emaciation and cachexia have supervened—"ragged-edge" cases. Having no true contraindications, it may be used and by all means should be chosen when such conditions as acute or subacute nephritis, bronchitis, and active pulmonary phthisis exist; conditions which add definitely to the dangers of chloroform and ether. While not, as has been said, "contraindicated" in any true sense, nitrous-oxide oxygen is usually unsatisfactory for any prolonged anesthesia, in the big, rugged, muscular "outdoor" type, such as laborers and athletes. Alcoholism—chronic—renders it ineffective; as a rule, the frequent result of an attempt to anesthetize being to produce only a violent delirium beyond which gas seems incapable of "taking" the patient. This may be overcome usually by a large dose of morphine half an hour before anesthesia is induced.

It must not be taken that $N_2O$-O is not feasible for abdominal work; on the contrary, we know of several clinics where it is extensively so used, but the surgeon who has always been accustomed to the very deep narcosis of chloroform or ether must learn to adapt himself to different conditions; in fact, in adopting gas as the anesthesia, he finds it necessary to almost entirely alter his "style" of operating. First, he finds that he must accustom himself to working through comparatively rigid muscles. If he has never used one before, he will probably insert a self-retaining retractor. He must learn to handle the abdominal viscera with a deft gentleness free from trauma and insult. He must not "yank around" or paw over the guts and omentum. Under the light $N_2O$-O anesthesia his patient will resist with a breath-holding and resultant straining that is subconsciously voluntary, and that, should the rough tactics be continued, will obviously be followed by shock. And he must have at the head of the table a man trained in the use of $N_2O$-O and accustomed to its frequent administration. A tyro can give chloroform or ether—after a fashion—at least such an one can give an anesthesia under which the surgeon may work, but the giving of a good nitrous oxide anesthesia or analgesia is an art acquired only by practice, and full knowledge of this agent's action, good judgment in case selection, and a thorough familiarity of the physical characteristics of compressed $N_2O$-O gas and the apparatus for its administration. Of the latter there are many, and to my belief they are all good. I have used at least a half dozen different makes. To my mind there are but two absolute essentials to a good nitrous-oxide oxygen apparatus; reducing valves to insure a continuous gentle flow of the gas, and a mask properly designed to fit air-tight over the mouth and nose. The all soft rubber mask that comes in two sizes with one of the new machines is probably the most satisfactory to date, having certain advantages over the mask of rigid material with pneumatic rim.

This matter of air exclusion is frequently difficult as in the toothless, men with full beards, and patients with extremely long, narrow faces, but air exclusion is essential to good gas anesthesia.

Recapitulating then on the field and limitations of nitrous oxide in surgery, we can only roughly classify the cases in which it is applicable. Many considerations are to be weighed and each case must be treated individually as to the selection of the anesthetic. Roughly for all work away from the abdomen, where quiet unconsciousness suffices, and deep relaxation is not required, for example, thyroidectomy, breast amputations, mastoid, etc. For all cases where there exists a definite, danger-increasing contraindication to chloroform or ether. For the "ragged-edge" cases referred to above, for all short and minor procedures—an ideal office anesthesia.

It has been found in the last few years that $N_2O$ diluted with a greater percentage of oxygen than is the case when given for complete anesthesia, will produce, and indefinitely maintain, so long, of course, as it is continuously given, an almost ideal analgesia; that is, a condition in which consciousness is not lost; but pain perception is obliterated; a dazed "groggy" condition during which the patient can respond to suggestions and commands, but of which there is afterward no memory. This amnesia suggests that the everday working mind, as it were, goes out of commission and the subconscious mind comes forward to take its place. The campaign or propaganda waged in the secular press so enthusiastically some years ago for the Freiberg "Twilight Sleep," which now, I think I am safe in saying, has been pretty generally discredited, created a demand for a "Painless Childbirth," and it occurred to various obstetricians and anesthetists that $N_2O$-O had here a field. The idea was not new, gas and oxygen having been used by Klikowitsch in Russia as long ago as 1880. But for various reasons its use had not spread from this beginning, nor had occasional sporadic revivals of it established it. When, however, Guedel of Indianapolis, Lynch, Davis, Webster, and others began to advocate the method and report cases six or seven years ago, and when later "Twilight Sleep" was, after trial, denounced by many, $N_2O$ analgesia seemed suddenly to take hold.

$N_2O$-O as applied to labor affords, all things considered, the nearest approach to an ideal "Painless Childbirth" that we have. That statement may be made without modification; but it does not mean that the method is perfect. None is, but in the cardinal features to be sought, viz.: safety (to both mother and child), efficiency, and general applicability it excels either the older inhalation agents, or any hypodermic hypnosis. The analgesic picture and its technic may be briefly described.

As has been said, we describe analgesia as loss of pain perception without loss of consciousness, or a marked dulling of the pain perception with, in either case, partial or complete amnesia, or loss of memory. Usually the actual pain, as described at the time, is negligible. Memory of it is very vague or entirely absent. There is a vast difference between analgesia and amnesia, and it is the difference between "Twilight Sleep" and the $N_2O$-O method. Pain will cause shock and exhaustion whether registered by the conscious or the subconscious mind, and the obstetrician who favors a morphine-scopolamine narcosis or hypnosis need but to see a $N_2O$-O analgesia to be drawn to the latter by contrast.

Assuming a normal labor, the exhibition of $N_2O$ may begin at any time. Usually through the first stage when the patient is up and about and the pains are not so frequent or so long, the patient, as yet showing no exhaustion and with courage and stamina unimpaired, will not ask for relief. At about the completion of the first stage then, when the patient goes to bed to remain, we usually start the gas, and a technic about as follows is used: The bag on the apparatus is two-thirds filled with $N_2O$, the patient is instructed to notify us on the first suggestion of a pain's approach. The mask is then placed over the face, and the patient's instruction is to take a few full, deep breaths. After about the third deep inspiration, a small quantity of oxygen is added to the $N_2O$, and this diluent is increased or diminished as conditions indicate. One may know only by experience what proportion of oxygen is correct. Of course, there must never at any time be even a suggestion of cynanosis or of jactitation or muscular spasm, and in the observance of this iron-clad rule for obstetrical analgesia with gas depends the safety of the unborn child. Gas, properly given, carries no danger to the infant in utero, but if the $CO_2$ content of the maternal blood stream is permitted for a prolonged period to remain way above normal the danger is obvious. After each three or four deep inhalations of the gas-oxygen the patient is instructed to hold her breath and bear down. This she will do readily, and with a little experience she will make more rapid progress through the second stage. Finding that her voluntary efforts do not cause her pain, she will quickly learn to work the harder under the analgesia—she will not shirk her own part, as is so frequently the case when dread of the increased pain is uppermost in her mind. As the contraction subsides the gas is withdrawn, and the patient relaxes, often with a smile. She frequently recounts a dream, and, if the analgesia has been proper, has no memory of pain. The procedure described above is repeated with each contraction, until toward the last, when the pains are expulsive in character, are practically continuous and the head is well down on the perineum, the analgesia is merged into light anesthesia and the actual delivery is effected during this time. The patient may be held anesthetized while the perineal examination is made and while the repair, if necessary, is done, or she may be permitted to awaken, and reanesthetized for the placing of sutures.

Any operative or manipulative procedure may be carried out under gas. I have given $N_2O$-O analgesia for a forceps delivery. The patient was conscious of the application of the blades, of each contraction, and of every time traction was made; she was conscious of the actual delivery but had afterward only a hazy recollection of the procedure and said she experienced no pain, but that she recalled it as a dream. Ordinarily, of course, anesthesia—complete unconsciousness—is preferable to analgesia for manipulative work, but the above is cited merely as an example of the analgesic state, as produced by gas, when ideal.

There will occasionally be encountered an obstetrical patient of a nervous or hysterical type in whom a satisfactory analgesia cannot be produced because a violent delirium supervenes as the first effect of two or three inhalations. This intoxication, which is the "stage of excitement," is sometimes observed only with the first two or three attempts and by suggestion can finally be overcome, but most frequently the only method of overcoming it is to induce an anesthesia with each pain,—for total unconsciousness will of course control the delirium,—change to another agent, or worry along until toward the last when light anesthesia can be maintained for the delivery and the powerful expulsive contractions immediately preceding it. Nitrous oxide differs from ether or chloroform in this: that the pains are not checked even when full anesthesia is induced. The musculature of the uterus is not paralyzed and the contractions take place with apparently the same frequency and strength. There is, of course, this difference—and it explains why analgesia is preferred to anesthesia—that the contractions cannot be augmented by the *conscious* bearing down efforts; and this assistance, as we all know, is invaluable.

Recapitulating for conclusion: First, $N_2O$-O is the safest agent we have for the production of a so-called "Painless Childbirth." It is eliminated solely by the lungs, and immediately upon its withdrawal. It puts no additional strain upon heart, lung or kidney and is totally without late or secondary dangers or complications. If properly given, the danger to the child is not one whit increased. Babies born under $N_2O$-O analgesia or anesthesia cry as readily as those born without. We have seen no difficulty with delayed animation, and no still-born infants, or babies dying in utero during labor.

Second. Should full anesthesia be induced, the uterine contractions continue as before.

Third. In the large majority of cases the effect is pleasant to the patient, and eminently pleasing and satisfactory. I have never seen a mother whose comparison of $N_2O$ analgesia with any other method she might have had was not decidedly favorable to the former.

Fourth. Safety and efficiency depend upon the skill with which it is given, and the ability and experience of the anesthetist.

Fifth. It is as suitable for the residence as for the hospital. No special surroundings for "psychic effect" are needed. It is the nearest approach to the ideal "Dammerschlaf," "Twilight Sleep" or "Sunrise Slumber."

